# Retargeting the *Clostridium botulinum* C2 toxin to the neuronal cytosol

**DOI:** 10.1038/srep23707

**Published:** 2016-03-30

**Authors:** Benjamin J. Pavlik, Elizabeth J. Hruska, Kevin E. Van Cott, Paul H. Blum

**Affiliations:** 1 Department of Chemical and Biomolecular Engineering, 207 Othmer Hall, University of Nebraska-Lincoln, Lincoln, NE 68588-0643, USA; 2 School of Biological Sciences, 1901 Vine Street, University of Nebraska-Lincoln, Lincoln, NE 68588-0665, USA

## Abstract

Many biological toxins are known to attack specific cell types, delivering their enzymatic payloads to the cytosol. This process can be manipulated by molecular engineering of chimeric toxins. Using toxins with naturally unlinked components as a starting point is advantageous because it allows for the development of payloads separately from the binding/translocation components. Here the *Clostridium botulinum* C2 binding/translocation domain was retargeted to neural cell populations by deleting its non-specific binding domain and replacing it with a *C. botulinum* neurotoxin binding domain. This fusion protein was used to deliver fluorescently labeled payloads to Neuro-2a cells. Intracellular delivery was quantified by flow cytometry and found to be dependent on artificial enrichment of cells with the polysialoganglioside receptor GT1b. Visualization by confocal microscopy showed a dissociation of payloads from the early endosome indicating translocation of the chimeric toxin. The natural *Clostridium botulinum C2* toxin was then delivered to human glioblastoma A172 and synchronized HeLa cells. In the presence of the fusion protein, native cytosolic enzymatic activity of the enzyme was observed and found to be GT1b-dependent. This retargeted toxin may enable delivery of therapeutics to peripheral neurons and be of use in addressing experimental questions about neural physiology.

Naturally occurring neurotoxins have long been used to study neural physiology, and the exploitation of modified biological neurotoxins as drug delivery systems is expanding^1^,^2^. These toxin-based delivery systems are multi-domain proteins that bind target cells and translocate material (payloads) across the lipid bilayer into the cytosol of the targeted cell. These systems are altered AB-type toxins, consisting of a payload domain (A) and a binding/translocation domain (B). The A and B domains can be covalently linked by a polypeptide or disulfide bond that is later cleaved during the translocation step^3^,^4^,^5^,^6^. Non-covalently linked (binary) A and B toxin domains are transcribed and translated independently and associate prior to exerting toxicity. These binary systems have recently been studied in the context of payload delivery to cancer cells^7^. It is advantageous from a protein engineering perspective to design separately expressed molecules because binding/translocation and payload modules can then be developed independently. The *Clostridium botulinum* C2 toxin (C2) is not a neurotoxin, but it has a binary AB toxin design and been shown to deliver a variety of engineered payloads in a nonspecific manner to a variety of cells^8^,^9^,^10^,^11^. It was not known if the binary AB-type C2 toxin structure could be used as a platform to introduce a new binding specificity and deliver molecular payloads. Here it was hypothesized that by replacing the C2 toxin binding domain with a *C. botulinum* neurotoxin (BoNT) serotype C1 binding domain (C1 H_CC_), the engineered B domain and payload could be expressed separately, combined and enable targeting of *neural* cells, while preserving the normal C2 translocation process.

The native C2 toxin is composed of two separate proteins. The B domain protein (C2II) binds target cells and translocates the A domain (C2I, the payload). The A domain is an ADP-ribosyltransferase that causes cell rounding and apoptosis initiated by ADP-ribosylation of cytoplasmic actin^12^,^13^,^14^,^15^ (Fig. 1a). C2II monomers are proteolytically processed to remove a 20 kDa segment from the N-terminus, which activates the binding/translocation domain into C2IIa^16^. C2IIa monomers then spontaneously oligomerize and bind the cell surface via interactions with asparagine-linked glycans on the cell membrane^17^,^18^. The A domain, C2I, binds to the C2IIa oligomers and the C2IIa/C2I complex is internalized by clathrin and Rho-dependent mechanisms^17^,^19^,^20^. Acidification of the early endosome causes membrane pore formation by C2IIa oligomers, through which C2I is transported into the cytoplasm^21^,^22^.

Generally, modification of toxin binding specificity is accomplished by the incorporation of a heterologous protein domain with concurrent ablation of native binding affinities by mutagenesis^7^ or complete replacement of the binding domain^23^. Blocker *et al.* showed that truncating the C-terminal binding domain of C2II by seven amino acids or removing of the entire binding domain maintains the stability of C2IIa and allows for oligomerization, but prevents receptor binding^24^. This led here, to the proposal that the C2 binding domain could be engineered to confer a new target cell binding specificity by replacement of the C2 C-terminus with another toxin–derived C-terminal binding domain (Fig. 1b). BoNT heavy chain C-terminal binding domains are generally similar in size to C2 binding domain, belong to the beta-trefoil fold family (indicating structural stability), and have a high neurological binding specificity^25^,^26^,^27^. Natural binding targets of BoNTs are peripheral presynaptic cholinergic neurons at the neuromuscular junction^28^. Complex gangliosides such as GT1b are enriched in these neurons and are required for certain BoNT serotypes to target and intoxicate cells^29^. BoNT C1 does not have an identified protein receptor and endocytosis has been shown to be ganglioside dependent^30^.

It was not known previously if retargeting of the C2 toxin by replacement of its binding domain would be structurally and functionally compatible with activation, oligomerization, binding, and translocation. Here the entire binding domain (~15 kDa) from the B component of C2 was removed and the C1 H_CC_ binding domain was inserted using a rigid polypeptide linker. The resulting fusion protein oligomerized *in vitro* after activation by trypsin and delivered a fluorescently-labeled payload and the native C2 cytotoxic enzyme to multiple neural and non-neural cell lines enriched with the C1 H_CC_ receptor trisialoganglioside GT1b. The results presented here demonstrate that an engineered C2II-based molecule can be redirected to bind to specific cell populations and translocate/deliver protein payloads. Future engineering of payloads may enable development of new methods to study and visualize intracellular biology.

## Results

### Fusion protein expression and characterization

Multiple recombinant protein constructs were expressed and purified using *E. coli* that were based on the BoNT serotype C1 neurotoxin and the ADP-ribosylating C2 toxin. The native BoNT C1 is depicted in Fig. 2a, and the native C2II binding/translocation domain is depicted in Fig. 2b. C2 toxin with C-terminal deletion of domain 4 (C2IIΔD4)^24^ and the C1 neurotoxin binding domain C1 H_CC_ were produced for use as controls. The C2IIΔD4 and C1 H_CC_ of BoNT C1 (1094–1291) were linked with a glutamate-proline ten-repeat peptide linker (EP)_10_ to generate C2II-C1 (Fig. 2c). In addition, two C2I-based payloads were constructed including a non-toxic C2It (1–226) that excludes the active enzyme site and a full length C2I (1–431) (Fig. 2d).

Cleavage of the glutathione affinity tag (GST) and activation of C2II-C1 by trypsin into oligomers is shown in Supplementary Fig. S1. *E. coli* BL21(DE3) cells were lysed and ultracentrifuged to remove insoluble proteins and the supernatant (lane 1) was passed over the affinity resin. The resin was then washed extensively, and protein-bound resin was loaded into lane 2 to examine the mass of the full length resin-bound protein and the extent of thrombin cleavage. The resin was then treated with thrombin to cleave the GST tag (resin and supernatant were loaded into lane 3). Proteins were then eluted from the resin and treated with trypsin. The trypsin-activated C2II-C1 monomers oligomerized as indicated by a shift in electrophoretic migration from an observed mass of ~90 kDa to a much greater mass than 250 kDa. Activated C2IIΔD4 was also produced with the same method and compared to activated C2II-C1. The heptameric form of C2II-C1 had an expected molecular mass of ~497 kDa, and heptameric C2ΔD4 had as an expected molecular mass of ~350 kDa. The C2II-C1 oligomer had a higher mass than that of C2IIΔD4 oligomer, as expected. The oligomerized forms of C2II-C1 and C2ΔD4 maintained stability in SDS during electrophoresis as previously reported^24^ and dissociated partially with the addition of heating (Supplementary Fig. S3). An additional band was identified during purification with anti-BoNT C1 antigenicity (Supplementary Figs S1 and S2). However, after extensive heating of C2II-C1 oligomers it was determined the dissociated composition was predominantly of full-length C2II-C1 monomers (Supplementary Fig. S3).

Western blotting was then conducted of oligomerized C2II-C1 and C2IIΔD4 (Supplementary Fig. S1). Proteins were probed with an anti-BoNT C1 antibody. BoNT C1 H_CC_ (MW ~23 kDa) was used as a positive control. C2II-C1 oligomers cross-reacted with the anti-BoNT C1 antibody, while the oligomerized C2IIΔD4 did not cross-react. This confirmed that BoNT C1 H_CC_ was successfully fused to C2IIΔD4 via the (EP)_10_ repeat linker in the oligomeric state.

### Neural targeting of fluorescently labeled C2It payloads by C2II-C1

The binding and payload internalization that was mediated by the C2II-C1 binding/translocation component was investigated using a fluorescently labeled C2I-based payload, C2It (Fig. 2d), to populations of cells with and without the GT1b ganglioside receptor. Murine neuroblastoma Neuro-2A (N2A) cells do not naturally present GT1b on the cell surface, but can be artificially enriched^30^. After payload C2It was purified (Fig. 3a), an Alexa Fluor 488 succinimidyl ester label was conjugated to the protein (C2It-488). C2It and activated C2II-C1 were incubated with differentially GT1b-enriched N2A cells. After removal of extracellular proteins by enzymatic digestion, flow cytometry was used to quantitate internalized C2It-488. The highest number of cells with increased fluorescence corresponded to GT1b enrichment and addition of C2II-C1 (Fig. 3b panel 2). Background uptake of C2It without C2II-C1 in the presence and absence of GT1b was minimal. Enrichment alone with GT1b did not give significantly increased background fluorescence (Fig. 3b panel 1,4). Uptake of the C2It-488 payload alone was also minimal (Fig. 3b panels 3,6). The highest non-target uptake (~2%) occurred in the population with C2II-C1 and C2It without GT1b (Fig. 3b panel 5). Student’s t-test determined that the dependence of C2It-488 uptake on C2II-C1 and GT1b was significant with *p*-values < 0.05 between experiments. Overall, an intracellular C2It-488 delivery efficiency of ~18% (percentage of cell population) was achieved in the presence of GT1b and C2II-C1 (Fig. 3c).

After quantitation of binding and internalization by flow cytometry, C2It delivered by activated C2II-C1 to targeted cells was visualized by confocal fluorescence light microscopy to determine intracellular localization. C2It was conjugated to an Alexa Fluor 568 fluorescent dye (C2It-568). Channel separated imaging was conducted of C2It-568 (red), Rab5a-GFP early endosomal marker (green), and DAPI nuclei (blue). It was observed that an intracellular C2II-C1-delivered C2It-568 colocalized at a low level with early endosomes when cells were enriched with GT1b (Fig. 4a). This result was consistent with endosomal escape of C2It by an active translocation domain. Without GT1b, C2It-568 signals were confined generally to the outside of the cell with low levels of reporter associated with early endosomes (Fig. 4b). These findings are consistent with the binding/internalization flow cytometry data (Fig. 3b,c). Additional control permutations lacking C2II-C1, GT1b or C2It-568 did not achieve intracellular delivery of C2It reporters with early endosomal dissociation (Supplementary Fig. S5).

### Retargeting of the native C2I enzyme by C2II-C1

Delivery of an active enzyme to the cytosol was determined by cell rounding caused by native C2I payload in both human glioblastoma A172 and synchronized HeLa cell lines differentially enriched with GT1b. Full length C2I was purified (Fig. 5a), combined with activated C2II-C1, and then added to cell line cultures for seven hours. Cell rounding was determined to be 2.8-fold higher than the non-GT1b enriched A172 cell population (Fig. 5b). Delivery-dependent cell rounding of synchronized HeLa cells was investigated as a non-neural cell line enriched with GT1b for a comparison to previous data of the wild type C2II without GT1b enrichment previously reported by Barth *et al.*^31^. Flow cytometry methods confirmed the synchronization of HeLa cells in the early S-phase by quantitation of DNA (Fig. 5c). In synchronized HeLa cells, rounding was 2.1-fold above the non-GT1b enriched population. (Fig. 5d). Student’s t-test was used to evaluate the experimental significance between experiments. Comparing control populations to the GT1b-enriched population produced *p*-values < 0.05.

## Discussion

Delivery of molecular payloads to the cytosol of actively targeted cells has been a long standing goal in molecular medicine and biochemical research^32^. Bacteria have evolved toxins to target cells and deliver toxic payloads to the cytosol; they are now being engineered to instead deliver beneficent payloads^33^,^34^. In general, there are two classes of AB-type bacterial toxins: linked and unlinked (binary). Linked toxins consist of a single chain protein having both a toxin domain and a binding/translocation domain. Binary toxins consist of two separately expressed protein molecules, where the binding/translocation domain and the toxin domain assemble *via* non-covalent interactions. For therapeutic development, engineering of a binary toxin offers advantages because the binding/translocation domain and the payload domain can be separately expressed and purified. The C2 toxin from *C. botulinum* is a binary structure, but is nonspecific as it binds a variety of cells and necessitates N-linked glycans for intoxication (*i.e*., it is not a specific neurotoxin)^35^. The approach pursued in this study was to engineer the C2 toxin binding domain by retargeting it to neural cells. This was achieved using a binding domain from the C1 botulinum neurotoxin, which has been previously applied as a targeting component for drug delivery to peripheral neural tissue in *linked* toxin designs and as liposomal surface modifications^2^,^36^,^37^.

Binding domain replacement of the C2 toxin requires that the retargeted binding/translocation component retains its ability to oligomerize upon activation, bind to the new targeting moiety on the cell surface, and translocate the payload into the cytosol of the target cell. The natural binding domain of the C2 toxin is located at the C-terminal end of the molecule and is designated as D4 (see Fig. 2b). Previous findings have shown that D4 is not required for oligomerization, and that translocation pores can still be formed in artificial membranes if D4 is absent^24^. Therefore, D4 was deleted from C2II and replaced with a domain that would target the molecule to peripheral neurons: the BoNT C1 binding domain. The BoNT C1 H_CC_ (Fig. 2a) preferentially binds gangliosides GT1b and GD1b^27^,^38^,^39^. It has been found that BoNT/A N-terminal heavy chain domain (H_CN_) may assist in the orientation of the toxin for association with the membrane by interacting with phosphatidylinositol phosphates^40^. Our research shows that H_CN_ is not required for compatibility with C2-mediated translocation activity, indicating that although H_CN_ may be active in native BoNT translocation, it is not required in a chimeric C2II-C1 translocation event. Thus, the binding domain was taken from a linked toxin and inserted into the binding/translocation domain of a binary toxin. It was hypothesized that this would re-target the resulting molecule to neurons while maintaining the C2 toxin’s mechanism of activation and translocation. It should be noted that a similarity exists between BoNT and C2 endocytosis and translocation mechanisms in that a clathrin/rho/dynamin-mediated endocytic-endosomal entry pathway characterized by pH-dependent protein conformational changes is implicated for both toxins^19^,^21^,^41^,^42^,^43^.

Several attempts were made to express a soluble C2II-C1fusion protein that would oligomerize when activated with trypsin. Direct fusion of the C1 H_CC_ domain was not successful due to solubility problems. To remedy this limitation a flexible glycine-serine linker (G_4_S)_n_ was used but encountered similar issues. Finally, use of a rigid (EP)_10_ linker resulted in a soluble fusion protein that was compatible with activation and oligomerization (Supplementary Fig. S1). An (EP)_10_ linker was selected because linkers of the (XP)_n_ variety adopt linear conformations due to predominant *trans* X-proline configurations that improve the stability and activity of fusion proteins^44^,^45^,^46^. Furthermore, glutamate-proline dipeptide repeats have been previously expressed in *E. coli*^47^ and are not susceptible to trypsin cleavage compared to other rigid linkers such as (EAAAK)_n_. SDS-PAGE confirmed that the C2II-C1 fusion protein could be activated by limited trypsin digestion and then oligomerize. Western blotting was used to confirm that the C1 H_CC_ domain was incorporated into the oligomeric species. BoNT C1 antigenicity specific to the C2II-C1 oligomer and a decrease in electrophoretic mobility in comparison to C2II∆D4 demonstrated that C1 H_CC_ at the C-terminus of C2II-C1 did not prevent oligomerization and was compatible with limited trypsin digestion.

To quantify and visualize binding and internalization of a payload by C2II-C1, a fluorescently labeled C-terminally truncated C2I-based payload, C2It (Fig. 2d), was constructed for use in flow cytometry and microscopy experiments. C2It that was composed of amino acids 1–226 of C2I (not containing the ADP-ribosylating active site residues), was fluorescently labeled in two separate versions with Alexa Fluor 488 (C2It-488) and 568 (C2It-568) by amine reactive chemistry. Previously, BoNT C1 H_C_ entry was shown to be GT1b-dependent in N2A cells that were artificially enriched for GT1b^30^, and this strategy was adapted to study targeting by the C2II-C1 fusion protein. If the engineered B component, C2II-C1, were activated, oligomerized and associated with the fluorescently labeled A component, C2It, GT1b-dependent uptake of fluorescently labeled C2It should be observed. This cellular model does not employ electrostimulation as previously described to enhance BoNT C1 intoxication^48^ because entry alone was presumed to be sufficient for the non-neural-specific C2 component of the fusion to promote translocation activity. For flow cytometry, a culture of N2A cells was enriched with GT1b while another was not, both cultures were incubated with activated C2II-C1 and C2It-488, and then both cultures were treated with pronase to remove extracellular proteins prior being analyzed. Cells with intracellular fluorescence above 10^3^ absorbance units were counted by flow cytometry and repeated results showed that N2A cell populations enriched with the binding domain receptor GT1b preferentially took up C2II-C1-delivered fluorescent C2It (Fig. 3). The results shown here indicate that the BoNT C1 H_CC_ can be used to replace another toxin binding domain and result in a GT1b-dependent entry specificity, similar to that of the of the BoNT C1 H_C_ alone as presented by Karalewitz *et al.*^30^. To confirm this uptake was dependent on GT1b and to determine subcellular localization within N2A cells, confocal microscopy was employed (Fig. 4). C2It-568 preferentially entered GT1b-enriched cells and did not colocalize with fluorescently labeled early endosomes. Escape from the early endosome by transport of C2It through the pore created by the translocation domain is a determinant of payload delivery to the cytosol. These results are consistent with the expected association between the engineered payload and binding/translocation domain by GT1b-specific delivery of C2It by C2II-C1. Lack of colocalization of early endosomes with C2It-568 (Fig. 4a) provides evidence to pursue other payloads with the intent of cytosolic delivery to manipulate the cytosome.

To deliver an active enzyme to the cytosol by the C2II-C1 fusion, the native C2 toxin A component, C2I was produced. The C2I enzyme causes cell rounding in eukaryotic cells by ADP-ribosylation of cytosolic actin^31^,^49^. The effect of C2I was tested after delivery by C2II-C1 to human glioblastoma A172 and HeLa cell lines that were enriched with the ganglioside GT1b. A greater than two-fold increase in cell rounding of GT1b-enriched cell populations was found for both cell lines when compared to controls lacking GT1b enrichment. By comparison, payload-induced cell rounding of synchronized HeLa cells in the presence of the fusion translocator C2II-C1 was less efficient than reported by Barth *et al.* in the presence of the native C2II translocation domain^31^,^50^. Given the engineered structure of the retargeted C2II-C1 toxin and the difference in cell surface receptor, it is not surprising to have a different efficiency of delivery. A truncated form of C2II-C1 characterized during expression may have incorporated into C2II-C1 oligomers, which may result in a decrease in binding efficiency (Supplementary Figs S1–S3) Although an apparent lack of monomeric C2II-C1 in final purification fractions was evident by SDS-PAGE, it is possible that monomeric C2II-C1 dissociated or not incorporated into oligomers competed for binding with the functional form of the oligomeric delivery system. Overall, the finding confirmed the native cytosolic activity of the C2I enzyme specifically delivered by C2II-C1 in a GT1b-dependent manner.

**Figure 1:**
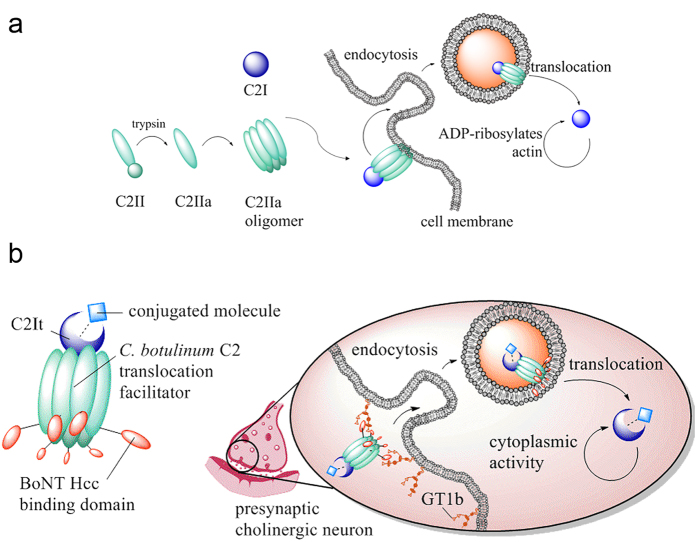
(**a**) Molecular steps of intoxication by the native C2 toxin. Heavy chain C2II requires protease activation and oligomerization to associate with C2I. After receptor mediated endocytosis, acidification of early endosomes causes C2I to be transported through the pore formed by C2IIa oligomers into the cytosol of the target cell to initiate its enzymatic activities. (**b**) Model of neural delivery based upon the C2II-C1 and C2It transport system. C2It is independently modified to carry a conjugated molecule as a payload for delivery mediated by activated C2II-C1. Targets in the cytosol of the peripheral nerve may be reached by use of this modular system. Payloads are limited by the requirements of conjugation and subsequent translocation by C2II-C1. Figure 1 graphics were generated by B.J.P using ChemBioDraw Ultra 14.0.

**Figure 2:**
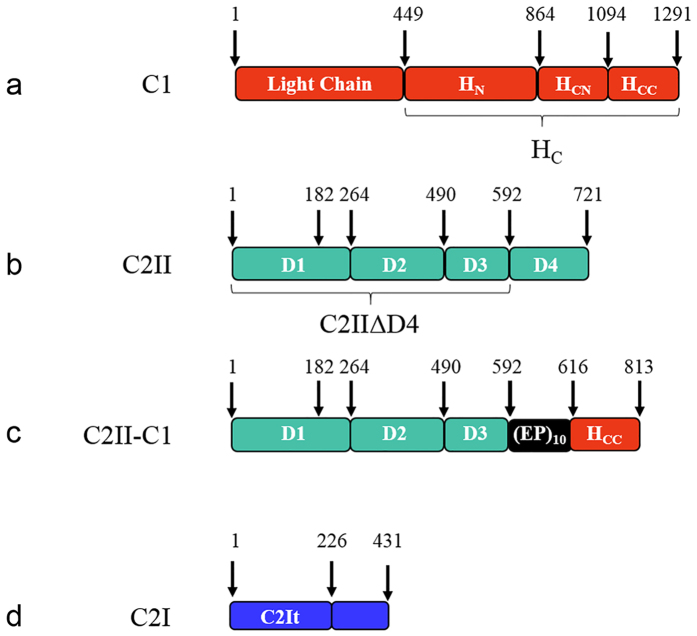
Protein domains of *C. botulinum* C1, *C. botulinum* C2II, fusion C2II-C1, and C2I. Numbers correspond to amino acid residues of each protein. (**a**) BoNT C1 has a linked enzymatic light chain payload and a binding/translocation domain. The heavy chain is separated into two regions, H_C_ and H_N_, with H_C_ further separated into N and C terminal domains H_CN_ and H_CC_. The H_CC_ was used in the C2II-C1 fusion construct (Fig. 2c) as the C-terminal binding domain. (**b**) The C2II binding/translocation component has four domains. Amino acid residue 182 indicates the trypsin cleavage position for activation of C2II into C2IIa. Domain 4 (D4) was removed to produce C2IIΔD4 as the translocation domain for C2II-C1. (**c**) The fusion C2II-C1 was made by linking C2IIΔD4 and BoNT C1 H_CC_ with an (EP)_10_ linker flanked by glycine-serine residue pairs on both sides. Amino acid 182 is the activation site for C2II-C1. (**d**) The native C2I enzymatic payload of the C2 toxin and truncated C2It domain. Amino acids 299, 348, 387 and 389 are essential for ADP-ribosylation activity of C2I^56^ and are therefore not present in C2It.

Alternate payloads based on modified C2It may be used in future delivery applications of the C2II-C1 fusion protein affecting the natural targets of BoNTs (Fig. 1b). A minimal region of amino acid residues 1–87 in the C2I component is required for complementary activity with the native C2II translocation domain^50^. Truncated C2I domains have previously been shown by fluorescent localization and cell fractionation to deliver biotinylated molecules to non-neurological targets such as epithelial cells, macrophages, and Jurkat T-cells by C2II^8^,^9^,^10^. Translocation of non-canonical polypeptides may also be possible with modified C2I, similar to payload development work recently conducted with anthrax lethal factor^51^. This work provides the basis of exploring other binding specificities and payload domains for additional applications.

## Materials and Methods

### Cloning of C2II-C1, C1 H_CC_, C2ΔD4, C2It and C2I

Plasmid pUC57-C2II-C1 H_CC_ was purchased as a codon-optimized gene synthesis product. It consists of the C2II gene truncated by seven C-terminal amino acids upstream to the C1 H_CC_ sequence, representing BoNT C1 amino acids Y1094-E1291. Primers C2IIΔD4F and C2IIΔD4-GS(EP)R amplified the gene corresponding to C2II amino acids M1-T592 and added a 5′ BamHI extension and 3′ glycine-serine-(EP) linking region to be used for overlapping PCR with the C1 H_CC_ domain. The BoNT C1 H_CC_ gene was PCR amplified with primers (EP)GS-C1 H_CC_F and C1 H_CC_R to contain a 3′ EcoRI restriction site. A second round of PCR was performed using GS(EP)10GSF and C1 H_CC_R to extend the 5′ amplicon of the C1 H_CC_ to complement the 3′ of the C2IIΔD4-GS(EP) sequence. The two resulting fragments were fused by overlapping PCR to yield C2IIΔD4-GS(EP)_10_GS-C1 H_CC_ (C2II-C1). To generate C1 H_CC_, PCR amplification was performed on the pUC57-C2II-C1 H_CC_ template using primers C1 H_CC_F and C1 H_CC_R. To generate C2IIΔD4, primers C2ΔD4F and C2ΔD4R were used to amplify the C2II gene without domain 4. Plasmid pUC57-C2It, was purchased as a codon optimized gene synthesis product. C2It (corresponding to C2I amino acids 1–226, PDB 2J3V) was directly subcloned into pGex-2T using BamHI and EcoRI restriction sites. Full length C2I (corresponding to C2I amino acids 1–431) was generated by overlapping PCR by fusion of C2It to DNA amplified from a synthetic DNA using C2IF and C2IR as flanking primers and C2IOF and C2IOR as overlapping primers. All final PCR products were digested by BamHI and EcoRI and ligated into pGex-2T. DH5α was transformed by electroporation to propagate C2II-C1, C1 H_CC_, C2IIΔD4, C2It and C2I as N-terminal GST fusions. DNA construct identities were confirmed with sequencing. Primer sequences are listed in Supplementary Table S6.

### Expression and purification of constructs

Fusion proteins were overproduced in *E. coli* BL21 (DE3). All cell lines were grown in 400 mL LB, 100 μg/mL ampicillin at 37 °C until induction at OD_600_ ~0.5 with 0.5 mM IPTG at 25 °C for 16 hr. Cells were harvested in 100 mL aliquots and the pellets were stored at −20 °C. Cells were resuspended in PBS, 1% Triton, pH 7.4, and a French press was used to lyse aliquoted cells by three passes at 10,000 psi. Cell debris was removed by ultracentrifugation at 80,000 × g for 20 minutes at 4 °C. Immobilized glutathione agarose (Genscript) was used to affinity purify GST fusion protein supernatants in batches using 150 μL of washed resin per 15 mL of culture supernatant and an incubation time of 1 hr at 4 °C. Resin was washed with PBS pH 7.4 to remove unbound protein. Proteins were cleaved from the GST tag according to manufacturer’s recommendations by bovine thrombin and separated from the purification resin by filtration using glass wool in a syringe. C2II-C1 was further processed by incubation with trypsin for 30 mins at a 1:5 enzyme to substrate ratio concluding with trypsin deactivation by trypsin inhibitor as described to activate recombinant C2II^21^.

### SDS-PAGE and western blot analysis

C2II-C1, C2IIΔD4, C1 H_CC_, C2I and C2It were separated by SDS-PAGE using a 10% polyacrylamide gel or by a 4–12% gradient Bis-Tris Gel (Supplementary Fig. S1) using methods described by Laemelli^52^. An anti-BoNT C1 polyclonal antibody (Metabiologics Inc., Madison, WI) was used to identify C2II-C1 using purified C1 H_CC_ as a positive control and C2IIΔD4 as a negative control. Proteins were separated by SDS-PAGE, transferred to a nitrocellulose membrane in Towbin buffer, blocked with 5% powdered milk in PBS-tween buffer and then probed with a 1:5,000 dilution of a 1μg/ul anti-BoNT C1 antibody in 0.5% powdered milk in PBS-tween, similar to methods by Towbin and Burnette^53^,^54^. Anti-rabbit HRP secondary antibody in 0.5% powdered milk, PBS-tween (1:5,000), was used for signal detection with ECL blotting substrate. Western signal images were developed on film. Gels and western final images were generated with UVP EC3 Imaging system and UVP Visionworks software.

### Cell culture of neuro-/glioblastoma and HeLa with ganglioside GT1b enrichment

Neuro-2a cells (N2A) (ATCC, CCL-131) were cultured in Eagle’s minimal essential medium (EMEM) supplemented with 10% (v/v) fetal bovine serum (FBS) and penicillin-streptomycin. A172 cells were grown in DMEM supplemented with 10% (v/v) FBS and penicillin-streptomycin (100 U/mL-100 μg/mL). HeLa cells (ATCC, CCL-2) were cultured in EMEM supplemented with 10% FBS and penicillin-streptomycin. HeLa cells were synchronized by double thymidine block with deoxycytidine release prior to ganglioside enrichment^55^. Ganglioside-enriched cells were prepared by sonicating 50 μg/mL GT1b (Enzo Life Sciences, Farmingdale, NY) in low-serum (0.5% FBS) culture medium for 20 min at room temperature. Cells were subsequently incubated 4 hr with GT1b^30^. Prior to addition of recombinant proteins, cells were washed three times with PBS to remove free ganglioside from the culture medium. Flow cytometry with a 488 nm laser line and 586/42 bandpass filter on a BD FACSCanto II was used to confirm HeLa synchronization by staining of DNA with propidium iodide. 10,000 cells/events were counted and statistical significance of average fluorescence per cell was determined by Student’s t-test (n = 3).

### Generation of payload C2It conjugates of Alexa Fluor 568 and Alexa Fluor 488

Amine reactive Alexa Fluor dyes were dissolved in anhydrous DMSO (10 mg/mL) and stored as aliquots at −20 °C. Purified proteins were concentrated to >5 mg/mL and adjusted to pH 8.5–9.0 with addition of 1 M sodium bicarbonate. Alexa Fluor in anhydrous DMSO was added to protein solutions with continuous stirring for 1 hr at room temperature. Excess Alexa Fluor and DMSO was removed by gel filtration (G-25 resin). Labeled proteins were ultracentrifuged at 80,000 × g and subsequently assessed for degree of labeling by spectrophotometry before and after ultracentrifugation. A degree of labeling greater than 1 fluorescent molecule per molecule of protein was used as a quality control cutoff and there was no visible pellet or appreciable change in spectrophotometric qualities after ultracentrifugation.

### Flow Cytometry of C2II-C1-delivered C2It-488 to N2A cells

**Figure 3:**
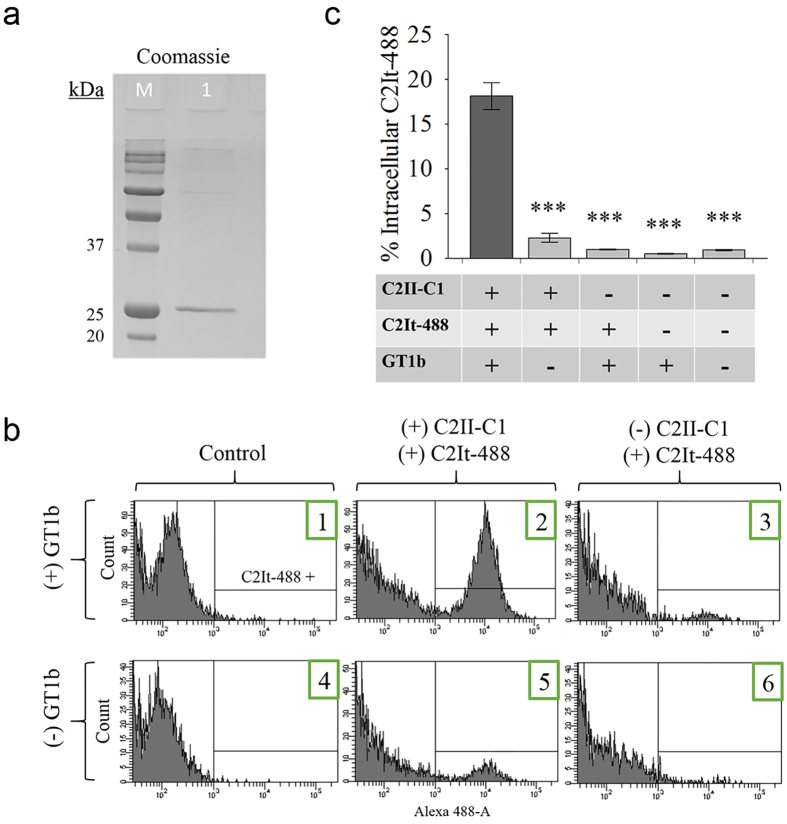
Flow cytometry to evaluate C2II-C1-mediated uptake of C2It-488 to differentially GT1b-enriched cell populations. (**a**) Coomassie stained SDS-PAGE of purified C2It (~26 kDa). Lanes: M: molecular ruler, 1: soluble elution fraction after thrombin cleavage. (**b**) Indicated N2A cells were GT1b-enriched and subsequently incubated with recombinant proteins activated C2II-C1 (2 μg/mL) and C2It-488 (4 μg/mL) for 2 hours. Cells were then processed with pronase (1 μg/mL) to remove membrane-bound C2It-488. Samples were analyzed by a BD FACS Canto II flow cytometer using FACSDiva software. (**c**) Quantitative assessment of intracellular fluorescence by flow cytometry. Percentages are expressed as a mean ± SEM (n = 3) and statistical significance of GT1b-dependent uptake of C2It-488 mediated by C2II-C1 was calculated using Student’s t-test by comparison to each control mean value. ***p < 0.005.

**Figure 4:**
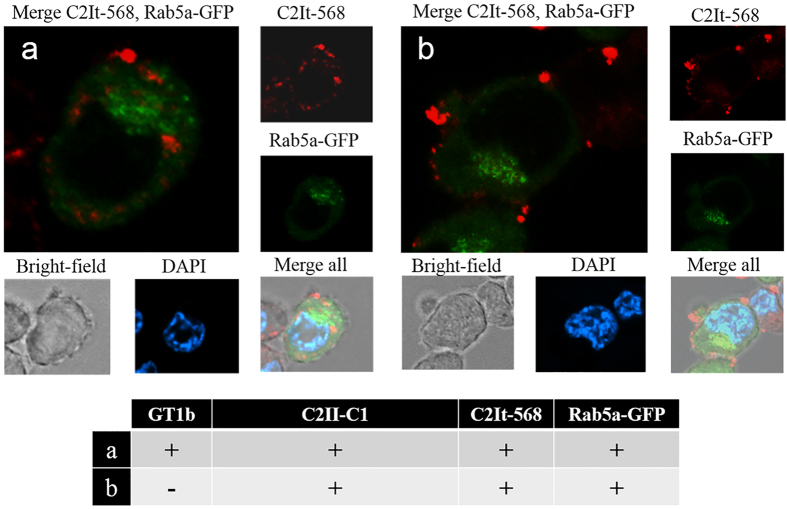
CLSM images of GT1b differentially GT1b-enriched N2A cells treated with C2II-C1 and C2I-568. All images were captured with 60x oil lens with 2x optical zoom. N2A populations were treated with a Rab5a-GFP early endosome marker (green) for 24 hr and stained with DAPI. Activated C2II-C1 (2 μg/mL) C2It-568 (red, 4 μg/mL) and GT1b (50 μg/mL) were incubated for 2 hours. (**a**) Cells were enriched with GT1b for 4 hr prior to addition of proteins. (**b**) Cells were not GT1b-enriched prior to addition of proteins.

**Figure 5:**
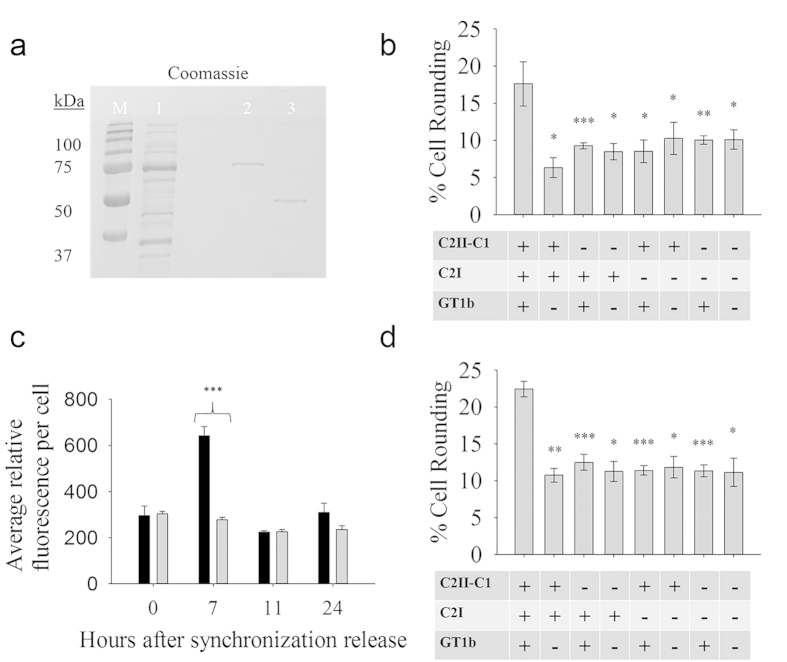
Cell rounding of differentially GT1b enriched cell populations by C2I mediated by C2II-C1. (**a**) Coomassie stained SDS-PAGE of purified C2I. Expected masses: C2I-GST (~75 kDa), C2I (~49 kDa). M: molecular ruler, 1: lysis supernatant, 2: purification resin prior to thrombin cleavage, 3: soluble elution fraction after thrombin cleavage. (**b**) A172 glioblastoma cells were grown to ~60% confluence and enriched as indicated with or without GT1b. Activated C2II-C1 (40 μg/mL) and C2I (20 μg/mL) were incubated with cells for 7 hr and observed for cell rounding. (**c**) Flow cytometry of synchronized HeLa cells stained with propidium iodide with and without release from thymidine block over time was used to confirm progression of S phase DNA synthesis after removal of excess thymidine and addition of deoxycytidine for release. (**d**) Cell rounding of differentially GT1b-enriched synchronized HeLa cells. Activated C2II-C1 (40 μg/mL) and C2I (20 μg/mL) were incubated for 7 hr and observed for cell rounding. All cell rounding percentages are expressed as mean ± SEM (n = 3) and statistical significance of cell rounding in the presence of GT1b, C2II-C1 and C2I was compared to controls using Student’s t-test. *p < 0.05, **p < 0.01, ***p < 0.005.

N2A cells were grown in 24 well culture plates to ~80% confluence. Cells were enriched with GT1b as indicated in Fig. 3c. Activated C2II-C1 was added at 4 μg/mL and C2It-488 at 2 μg/mL using a 0.5 mL working volume and incubated with cells for 2 hours. Cells were washed twice with PBS, then trypsinized and harvested. Cells were centrifuged and resuspended in PBS with pronase (1 μg/mL) and incubated on ice for 5 minutes. Protease inhibitor cocktail was then added and cells were centrifuged and resuspended in PBS with inhibitor cocktail. 10,000 events/cells were then counted by BD FACS Canto II flow cytometer using a 488 laser line and 530/30 emission band-pass filter. C2It-488 positive cells (greater than the absorbance threshold 10^3^ absorbance units) were counted and evaluated as a percentage of total cells. Replicated experiments were evaluated by Student’s t-test (n = 3).

### Fluorescence-based colocalization of proteins in Neuro-2a cell culture

Collagen-coated 12 mm no. 1 coverslips were placed into 24-well culture plates and seeded with N2A cells. N2A cells were grown to ~80% confluence. Purified C2It was labeled with Alexa Fluor 568 succinimidyl ester (C2It-568) instead of Alexa Fluor 488 to allow for discrimination from the early endosome marker. The baculovirus transduction system, BacMam 2.0 Cell Lights Rab5a-GFP early endosomal marker (Life Technologies), was added ~24 hr prior to GT1b enrichment. Cells were then enriched with GT1b as described in our methods. Recombinant proteins were added after washing of cells to remove free gangliosides. Activated C2II-C1 was added at 4 μg/mL and C2It-568 at 2 μg/mL using a 0.5 mL working volume and incubated with cells for 2 hours. Cells were washed with PBS, fixed with 4% paraformaldehyde and stained with DAPI. After processing, an Olympus Inverted IX-81 Microscope was used with an Olympus FV 500 confocal laser scanning microscope in sequence mode with laser lines 405 nm (blue), 488 nm (green) and 543 nm (red) to capture fluorescence images. Corresponding emission barriers used were 430–460 nm, 505–550 nm and 560–610 nm respectively. Transmitted light was used for cell morphology, and all images were captured using a 60x oil lens with 2X optical zoom. Contrast of all images was increased by 20%.

### Delivery of the native payload C2I by C2II-C1 to A172 cells and synchronized HeLa cells

Human glioblastoma A172 cells (ATCC, CRL-1620) were grown in 24 well culture plates to ~60% confluence to reduce cell rounding observed at higher confluence. HeLa cells were synchronized as described in the previous section. Both cell lines were enriched with GT1b as described in our methods at 50 μg/mL. C2II-C1 was added at 40 μg/mL and C2I was added at 20 μg/mL using a 0.5 mL working volume and incubated with cells for 7 hours. Pictures of cells were taken using an Amscope IN300TC inverted stereo microscope at 40x using Amscope MT v 3.0.0.5 software. Rounded cells were counted and determined as a percentage of total cells in the frame. The experiment was replicated three times and evaluated for statistical significance with Student’s t-test (n = 3). Institutional biosafety committee approval was obtained prior to execution of experiments with C2I in a biosafety level 2 laboratory due to anticipated toxicity when combined with C2II-C1.

## Additional Information

**How to cite this article**: Pavlik, B. J. *et al.* Retargeting the *Clostridium botulinum* C2 toxin to the neuronal cytosol. *Sci. Rep.* **6**, 23707; doi: 10.1038/srep23707 (2016).

## Supplementary Material

Supplementary Information
