# Supplementary material for: Retargeting the Clostridium botulinum C2 toxin to the neuronal cytosol
Source: Sci Rep. 2016 Mar 30;6:23707. doi: 10.1038/srep23707 (PMC4812341; doi:10.1038/srep23707)
Supplement: Supplementary Information [file srep23707-s1.pdf]

Supplementary Information

Retargeting the *Clostridium botulinum* C2 toxin to the neuronal cytosol

Benjamin J. Pavlik, Elizabeth J. Hruska, Kevin E. Van Cott, and Paul H. Blum

Figure S1. Characterization of recombinant proteins by SDS-PAGE and western blot. (a) SDS-PAGE of GST-purification of protein C2II-C1. Expected masses: GST-C2II-C1 (~117 kDa), C2II-C1 (~91 kDa), trypsin activated C2II-C1 monomer (~71 kDa), trypsin activated C2II-C1 oligomer (>>250 kDa). SDS-PAGE lanes: molecular ruler (M), 1: ultracentrifuged supernatant, 2: resin loaded before thrombin, 3: resin loaded after thrombin, 4: supernatant separated from resin after thrombin digestion. Arrows indicate the presence of a protein band doublet during purification in lane 4: i) full length C2II-C1, ii) truncated C2II-C1. **(b/c)** Oligomerization of C2IIΔD4 and C2II-C1 and anti-BoNT C1 reactivity. Samples of supernatant from GST-purification of C2II-C1 (**S1a**, lane 4) were activated by trypsin, separated by SDS-PAGE and visualized by Coomassie Blue G-250 **(b)** and western blot analysis with anti-BoNT C1 **(c)**. Purified BoNT C1 H_{CC} (~23 kDa) was used as a positive control for antigenicity. Lanes: M: molecular ruler, 1: activated C2II-C1, 2: activated C2IIΔD4, 3: BoNT C1 $H_{CC.}$

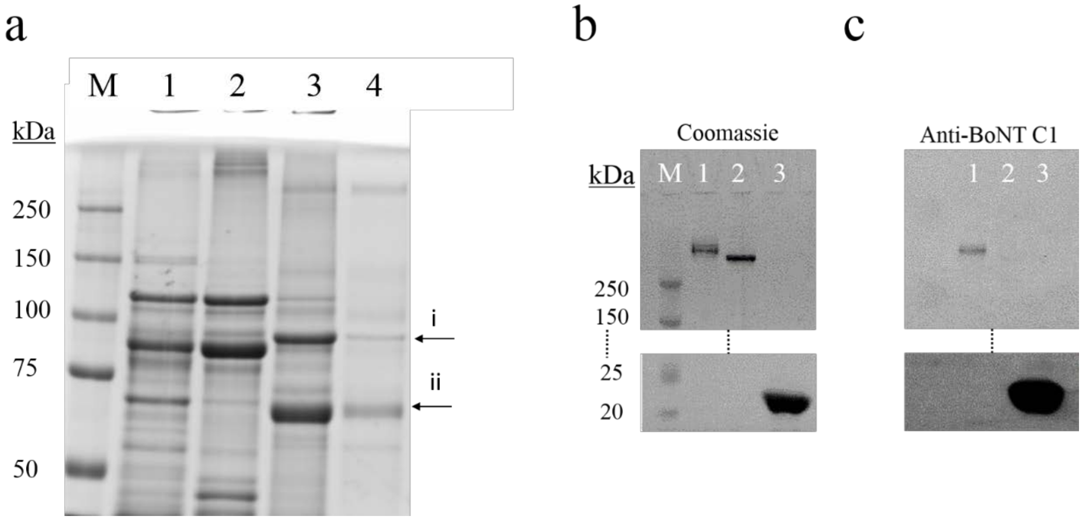

Figure S2. SDS-PAGE and anti-BoNT C1 western blot of C2II-C1 purification. This set of data is an extension of the purification gel from **Fig. S1a** to demonstrate the antigenicity of the doublet bands present during purification. Expected masses: GST-C2II-C1 (~117 kDa), C2II-C1 (~91 kDa), trypsin activated C2II-C1 monomer (~71 kDa), trypsin activated C2II-C1 oligomer (>>250 kDa). SDS-PAGE lanes: molecular ruler (M), 1: ultracentrifuged supernatant, 2: resin loaded before thrombin, 3, resin loaded after thrombin. Arrows indicate bands in the final elution fraction after thrombin treatment: a) full length C2II-C1, b) truncated C2II-C1, c) GST tag

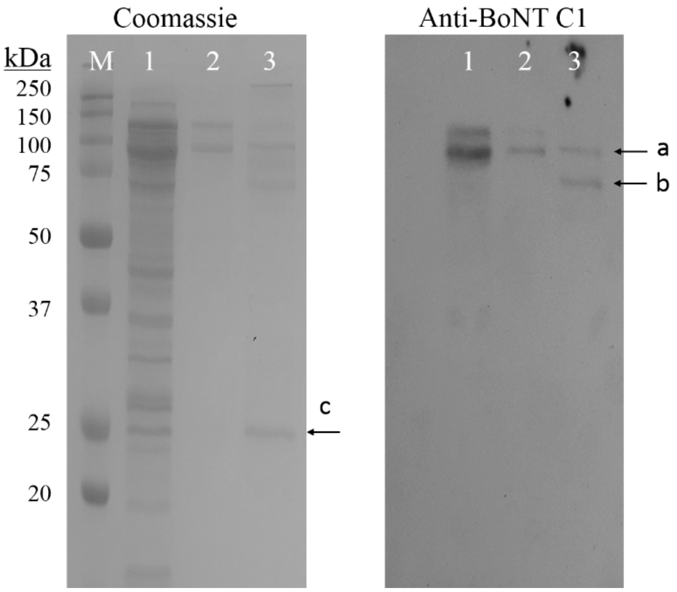

Figure S3. SDS- PAGE and anti-BoNT C1 western blot of extensively heated C2II-C1 oligomer. Expected masses: trypsin activated C2II-C1 monomer (~71 kDa), trypsin activated C2II-C1 oligomer (>>250 kDa). SDS-PAGE lanes: molecular ruler (M), 1: trypsin activated C2II-C1. A white arrow indicates the full length C2II-C1 monomer.

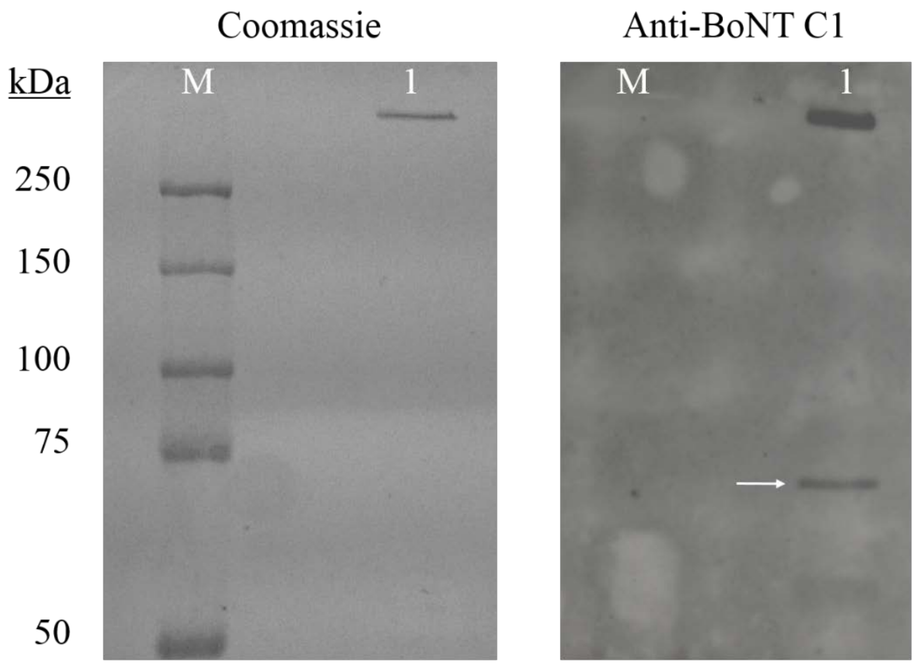

Figure S4. SDS-PAGE of activated C2II-C1 and C2ΔD4. These are full length versions of gels appearing in **Figure S1b,c.** Samples of trypsin-activated GST-purified C2II-C1 were separated by SDS-PAGE and visualized by **(a)** Coomassie Blue G-250 and **(b)** western blot analysis with anti-BoNT C1. Purified BoNT C1 H_{CC} (~23 kDa) was used as a positive control for antigenicity. Lanes: M: molecular ruler, 1: activated C2II-C1, 2: activated C2IIΔD4, 3: BoNT C1 $H_{CC.}$

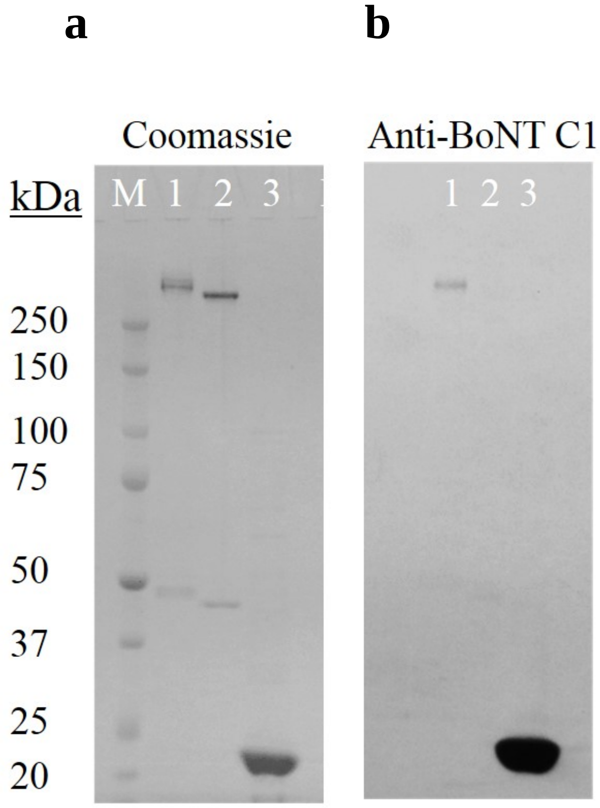

GT1b	C2ΔD4-$(EP)_{10}$-C1 H_{cc}	C2It/568	Rab5a/ GFP
-	-	+	+
-	+	+	+
+	+	+	+

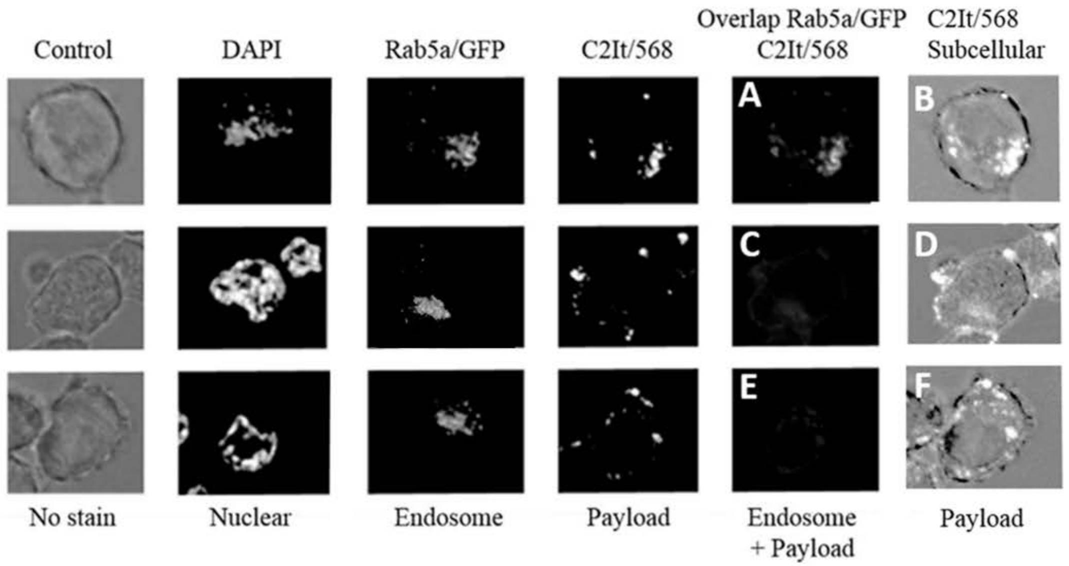

Figure S5. Channel separated localization of C2It-568 and early endosomes in N2A cell culture. A/B – Intracellular C2It/568 without GT1b or the C2II-C1 delivery unit co-localizes with the cellular periphery and/or early endosomes. C/D – Lack of co-localization of C2II-C1-delivered C2It/568 with early endosomes is consistent with a low intracellular signal when N2A cells are not treated with GT1b. The major abundance of C2It/568 in this treatment is confined to the cellular periphery. E/F – Intracellular presence of C2It/568 mediated by C2II-C1 in the presence of GT1b. Signals are not co-localized with early endosomes, indicating endosomal escape dependent upon delivery facilitated by C2II-C1.

Table S6. Primers for Cloning of C2IIΔD4-$(EP)_{10}$-C1 H_{CC} (C2II-C1), C1 H_{CC}, C2IIΔD4, and C2I. C2It was directly subcloned from synthetic DNA. C2It was extended by overlapping PCR to generate C2I. This extension of C2It was amplified from synthetic DNA prior to incorporation.

C2IIΔD4F	CGCGGATCCATGCTGGTCTCC
C2IIΔD4-GS(EP)R	CCGGCTCTGGTTCCGGTTCAGAACCGGTGATCACTTTGACCAGAATATTCATG
(EP)GS C1 H_{CC}F	CCAGAACCAGAGCCAGAACCAGGTTCTACCAACGTTGTCAAAGACT ATTGGGG
C1 H_{CC}R	CGGGAATTCTTATTCTGAAACCGGGAC
GS$(EP)_{10}$GSF	AACCGGAACCAGAGCCGGAACCGGAACCGGAACCGGAGCCAGAACCAGAGCCAGAACC
C1 H_{CC}F	CGCGGATCCATGGGCACCAACGTTGTCAAAGACTATTGG
C2IIΔD4R	CGGGAATTCTTA GGTGATCACTTTGACCAG
C2IF	CGCGGATCCATGCCGATTATTAAAGAACCGATTGACTTCATCAACAAACCGG
C2IR	CCGGAATTCTTAGATTTCTTTGTTTTGGATACCTTCAGCATCAAT
C2IOF	GCAAGAACTGGACTTTTACAACAAAGGCTCGGAAGCCTGGGGTGCGGAAAACTATG
C2IOR	CATAGTTTTCCGCACCCCAGGCTTCCGAGCCTTTGTTGTAAAAGTCCAGTTCTTGC
